# Optically Transparent and Highly Conductive Electrodes for Acousto-Optical Devices

**DOI:** 10.3390/ma14237178

**Published:** 2021-11-25

**Authors:** Alexey Osipkov, Mstislav Makeev, Elizaveta Konopleva, Natalia Kudrina, Leonid Gorobinskiy, Pavel Mikhalev, Dmitriy Ryzhenko, Gleb Yurkov

**Affiliations:** 1 Laboratory of EMI Shielding Materials, Bauman Moscow State Technical University, 105005 Moscow, Russia; konoplevaea@student.bmstu.ru (E.K.); natalia.kudrina1@gmail.com (N.K.); lgorobinskiy@bmstu.ru (L.G.); pamikhalev@bmstu.ru (P.M.); dsr@bmstu.ru (D.R.); ygy76@mail.ru (G.Y.); 2 N.N. Semenov Federal Research Center for Chemical Physics Russian Academy of Sciences, 119991 Moscow, Russia

**Keywords:** transparent electrode, mesh structure, sheet resistance, transparency, groove, lithography, chemical deposition, shielding efficiency

## Abstract

The study was devoted to the creation of transparent electrodes based on highly conductive mesh structures. The analysis and reasonable choice of technological approaches to the production of such materials with a high Q factor (the ratio of transparency and electrical conductivity) were carried out. The developed manufacturing technology consists of the formation of grooves in a transparent substrate by photolithography methods, followed by reactive ion plasma etching and their metallization by chemical deposition using the silver mirror reaction. Experimental samples of a transparent electrode fabricated using this technology have a sheet resistance of about 0.1 Ω/sq with a light transmittance in the visible wavelength range of more than 60%.

## 1. Introduction

Electrode element is an indispensable component of modern acousto-optical devices. It defines the shape of the acoustic field induced in the crystalline media [1,2]. Accurate apodization of the electrodes provides diffraction efficiency increase, higher apertures and better acoustic field homogeneity [3].

**Figure 1:**
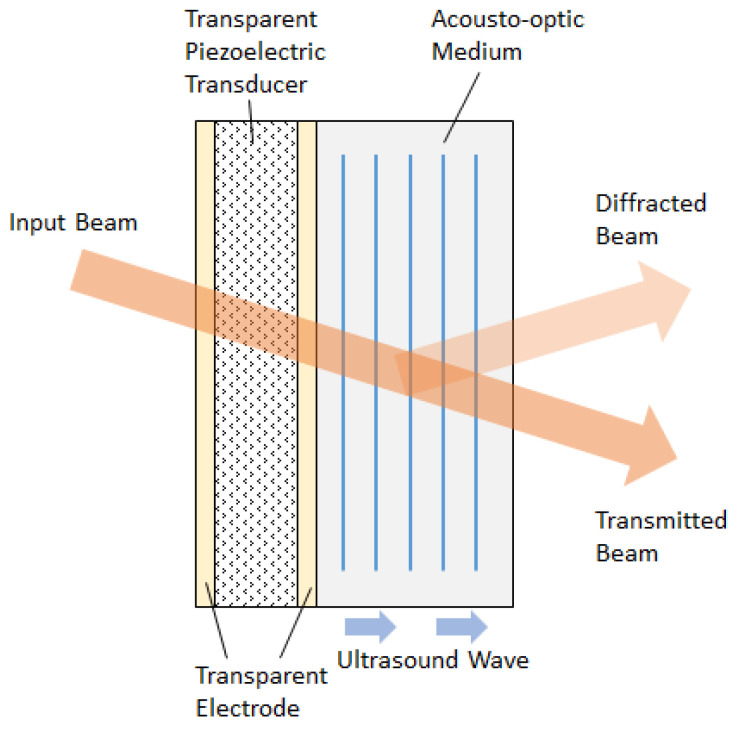
Design of a transparent acousto-optic modulator.

Transparent electrodes are necessary for acousto-optic devices, where the direction of light propagation coincides with sound wave (Figure 1). Such collinear geometry of acousto-optic interaction is widely used, for example, in acousto-optical tunable filters with high spectral resolution [4], which are necessary for Raman spectroscopy [5] and spectral-domain optical coherence tomography [6]. Propagation of light along ultrasound may be also potentially effective for multi-beam diffraction configurations [7] and other applications. Applying optically transparent electrodes in these devices may simplify its design and reduce its dimensions.

In conventional schemes of such electrodes, an ultrasonic wave may be formed by a transparent piezoelectric transducer based on ceramic materials with a perovskite structure (titanate-zirconate of a divalent metal (for example, lead), etc.) or polymer films (for example, polyvinylidene fluoride and its copolymers) with a thickness up to several tens of micrometers [8,9,10,11,12,13,14].

In addition, transparent electrodes are widely used in solar panels, touch screens, organic and inorganic diodes, etc. [15]. Among the approaches to the creation of such electrodes are the usage of transparent conductive oxides [15], metal nanowires [16,17], carbon nanomaterials [18], and metal micro- and nano-grids [19]. Today, thin films based on tin indium oxide (ITO) are the most widely used [20]. Still, their usage is limited by the low ratio of transparency and electrical characteristics for many current tasks; the best samples of ITO coatings have a surface resistance of about 20 Ω/sq with transparency in the visible range of about 90% [21]. In addition, this material is expensive due to the depletion of global indium reserves [22], and requires high-temperature annealing to obtain a high Q factors ϕ_s_ (ϕ_s_ = T_550_^10^/ρ_s_ [23]), which complicate the production of high-quality transparent electrodes on flexible polymer substrates. The latter is relevant due to the recent intensive development of flexible LCD touch screen technology [24,25].

A decrease in the electrode resistance leads to an increasing the transparent piezoelectric transducer efficiency. Additionally, electrodes with high electrical conductivity and transparency are necessary to increase the efficiency of solar cells or to increase electromagnetic protection and compatibility of electronic devices and equipment.

Among the approaches to creating high-electrical-conductivity and transparent structures, ordered metal mesh structures formed on the surface or inside a transparent substrate have considerable potential. The geometry of such systems can be calculated in advance and specified during manufacture. Electrodes based on these structures have the best characteristics at the moment [21] in terms of the ratio of sheet resistance and transparency (3–5 Ω/sq with transparency of more than 85% [19]).

Currently, several approaches are being developed to produce such microgrids [21,26]. One of the general approaches consists of forming microgrooves with a high aspect ratio in an optically transparent material, such as quartz, into which metal, for example, silver or copper, is subsequently deposited [22]. The grooves can be filled with metal by magnetron or electron beam deposition, or chemical deposition from the gas phase. However, due to the high aspect ratio and the small width of the groove, their use is problematic [22]. A rapid blockage of the inlet and the cessation of mental growth inside the grooves may occur during the deposition process. Similar problems are also typical for galvanic metal deposition into structures with a similar aspect ratio [27].

In this paper, the manufacturing technologies for transparent mesh structures are analyzed. A proposed method for filling the grooves produced by lithography methods with silver using the silver mirror reaction is also described. In addition, the article describes the results of studies of the deposited metal morphology and the measurements of transparency, sheet resistance, and the S parameters in the radio and microwave wavelength ranges in the open coaxial waveguide of the obtained structures.

## 2. The Analysis of the Production Technologies of Transparent Mesh Structures

There are three groups among technological approaches used to obtain ordered mesh structures:

- Approaches based on the formation of grooves of a specified geometry in a substrate or photoresist and their subsequent filling with metal. The grooves are formed using photolithography methods in combination with liquid or ion plasma etching or laser ablation;
- Approaches based on the removal of the preliminarily deposited metal layer using the same etching methods;
- Approaches based on additive processes (electrohydrodynamic printing).

**Figure 2:**
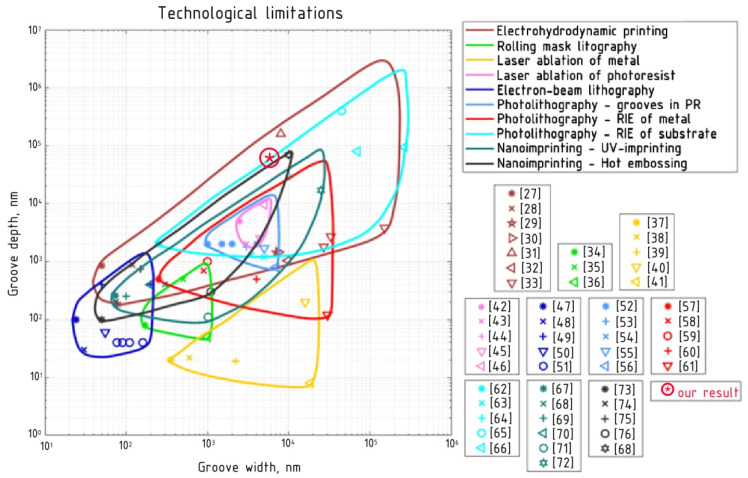
Technological limitations of various approaches to the formation of ordered mesh structures.

Based on the literature data [27,28,29,30,31,32,33,34,35,36,37,38,39,40,41,42,43,44,45,46,47,48,49,50,51,52,53,54,55,56,57,58,59,60,61,62,63,64,65,66,67,68,69,70,71,72,73,74,75,76,77], Figure 2 plots of the technological limitations of various approaches to forming grooves in transparent materials or directly forming conductors on the substrate surface. The markers show the values of the width and depth of the groove’s or line’s height, obtained from the literature sources, and the lines show the technological limitations of the approaches.

Two studies [78,79] provide a description of the calculated model and the results of the conductive mesh structures’ calculation, obtaining a screening coefficient SE of more than 50 dB with transparency of more than 90% in the visible range. These calculated values were obtained for a width and height of the mesh conductors of 1.5 and 14 µm, respectively. Per Figure 2, structures with such parameters can be produced by the following methods: forming grooves in a transparent substrate by photolithography followed by reactive ion plasma etching (RIE), nanoimprinting (hot embossing), or multilayer electrohydrodynamic (EHD) printing.

At this stage, the first method was chosen for the practical implementation of the calculated structures due to the wide availability of the necessary technological equipment and verified technological regimes. Notably, the methods of nanoimprinting and EHD printing, with their further development, have considerable potential for creating serial technology for producing large-area, micro-mesh, transparent electrodes.

## 3. Materials and Methods

**Figure 3:**
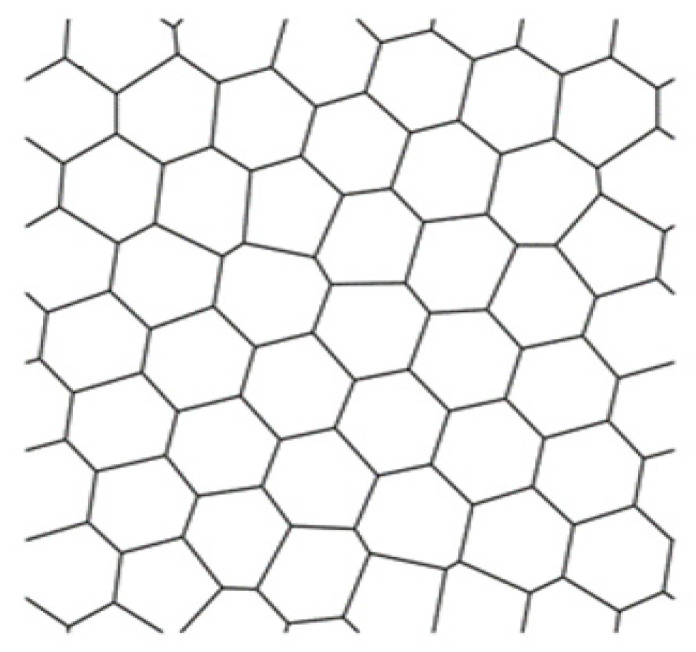
The topological figure of a conductive mesh.

Experimental samples were pure quartz wafers with a thickness of 0.5 mm. A system of grooves was formed in the first step of preparation as a network. In the second step, the grooves were filled with silver metal. The topological network was an artificial, disordered mesh with a Voronoi diagram randomly distributed inside a cell of equal probability density (Figure 3).

**Figure 4:**
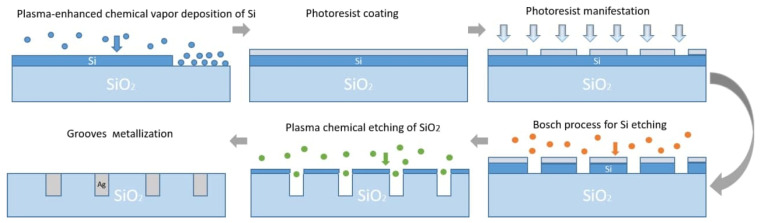
Technological process of micro-mesh formation.

The technological process of groove formation included these steps: the cleaning of a wafer, the coating of an α–Si:H masking layer with plasmochemical deposition using SPTS APM equipment (SPTS Technologies Ltd., Newport, UK); positive photoresist SPR700-1.0 coating by centrifuge, drying, and exposure with a ASML PAS 5500 stepper (ASML, Veldhoven, The Netherlands); followed by the development and plasmochemical etching of the α–Si:H masking layer with the Bosh process; and plasmochemical etching of grooves on the quartz wafer through the windows (holes) at the masking layer with the help of C_4_F_8_. (Figure 4).

A silver mirror reaction (a process of silver salt reduction, for example, silver nitrate) was used for filling the experimental samples with metal. The formation of a silver layer with few defects is possible only with a low rate of chemical reaction [80]. Glucose was used as a weak reducing agent, which increases silver adhesion to a surface [81]. The synthesis was carried out at 25 °C for the same reason.

Two solutions were used. The first solution was prepared by dissolution of 1.25 g of silver nitrate (AgNO_3_) in 30 mL of deionized water, the addition of 4 mL of 25% water ammonia (NH_4_OH), and the addition of 30 mL of an alkali solution (1.1 g of NaOH was dissolved in water and diluted until 30 mL of solution was formed). The initial rate of the silver mirror reaction decreased with increasing ammonia concentration; the silver nitrate solution stability and the thickness of the silver layer increased simultaneously [81]. The silver alkali ammonia solution was diluted to 100 mL, and the first solution was prepared. The second solution was prepared by dissolution of 1.1 g of glucose in water and dilution to 100 mL. A higher glucose concentration (higher than 1.3 g/L) decreases the maximal thickness of the silver layer [81] and facilitates silver particle agglomeration [24]. The shelf life of the solutions was less than 10 h. The samples for the metallization were placed into a beaker. The first solution was poured, and then the second solution was added in a volume ratio of 1:1. The reaction time was less than 10 min (usually 3–5 min). The long duration of the silver mirror reaction leads to the morphology of silver particles differing from a sphere [82], which can inhibit groove filling. The silver mirror reaction was repeated several times. The excess metal from the surface was removed after each iteration of silver deposition.

The geometric dimensions and surface morphology of the manufactured grooves and the surface morphology and structure of the deposited silver were studied by scanning electron microscopy (SEM, TESCAN ORSAY HOLDING, Brno, Czech Republic) using a VEGA 3. Imaging was performed at an accelerating voltage of 10 kV and a beam current of 10 A in secondary electron detection mode to obtain images with the highest resolution, and in back-reflected electron mode to obtain a compositional contrast.

Sheet resistance was determined by the four-probe method using a Keithley 2000 multimeter (Tektronix, Inc, Beaverton, OR, USA) and a Mill-Max 854-22-004-10-001101 four-probe head (Mill-Max Mfg. Corp., Oyster Bay, NY, USA).

The transmission coefficient in the visible wavelength range of 380 to 780 nm was determined on a Shimadzu UV-3600i Plus spectrophotometer (SHIMADZU CORPORATION, Kyoto, Japan) with a resolution of 1 nm at normal incidence of light on the sample.

Since the materials under development had considerable potential for application in radio engineering, the shielding efficiency (SE) was measured to evaluate the obtained materials’ radio engineering properties. The measurements were obtained in the frequency range from 10 MHz to 7 GHz on a specialized measuring stand based on a FieldFox N9916A vector circuit analyzer (Keysight Technologies, Santa Rosa, CA, USA) in a coaxial path (type II). The SE value was determined from the S21 transmission coefficient of the path with the sample in relation to the transmission coefficient S21 of the path without the sample. Electro-sealing gaskets composed of dense metalized fabric were used for better electrical contact between the sample and the walls of the coaxial tract. The SE dynamic measurement range of the stand is 80 dB and the measurement error is ±2 dB.

## 4. Results

**Figure 5:**
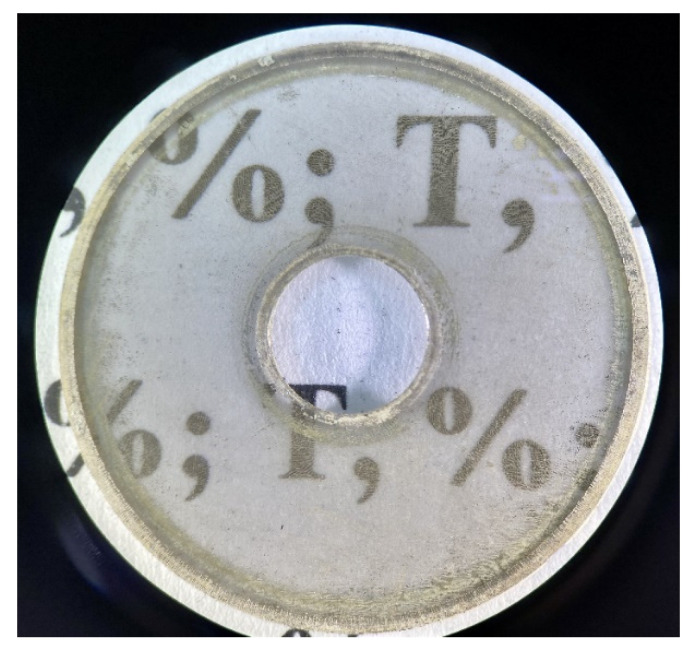
Photo of the manufactured sample.

A photo of an obtained sample is shown in Figure 5. The geometrical dimensions of the sample were an outer diameter of 16 mm and an inner diameter of 6.95 mm. The sample was fabricated in this shape due to the requirements for SE measurements in the coaxial path.

**Figure 6:**
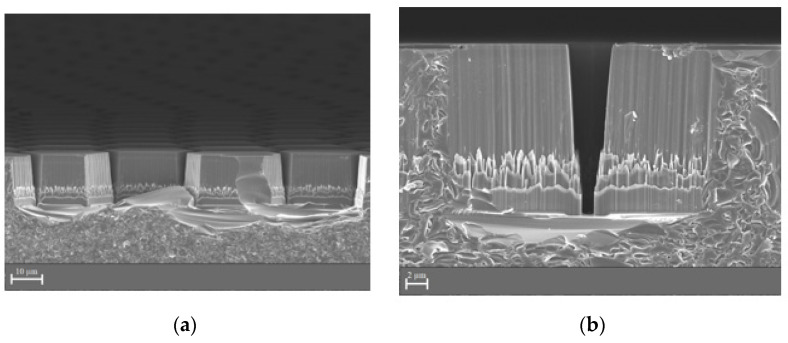
SEM images of the sample after the lithography and plasma chemical etching processes: (**a**) 2500× magnification; (**b**) 8000× magnification.

The grooves in the quartz substrate obtained by lithography and plasma chemical etching were examined by scanning electron microscopy (Figure 6). The groove walls obtained in quartz glass showed vertical deviations up to 10°, which led to distortion of the specified topology (Figure 3). The groove cross-section was trapezoidal with a depth of 16.24 µm and a width of 1.15 (in-depth) to 4.47 µm (at the surface). The deviation from the verticality of the groove walls was caused by lateral subtraction during plasma chemical etching.

**Figure 7:**
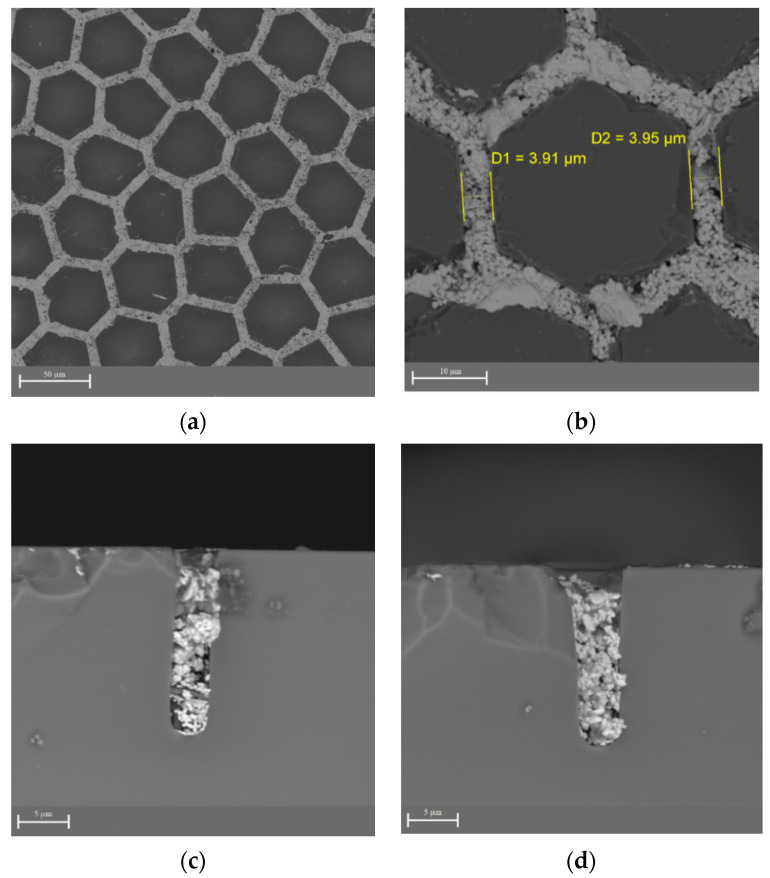
SEM images of the sample after metallization: (**a**) 1000×, (**b**) 4500×, (**c**) 7000×, and (**d**) 7000× magnification.

The degree of groove filling with silver was evaluated after the metallization process (Figure 7). We found that the silver filled all the grooves throughout the sample, but the silver structure was porous.

**Figure 8:**
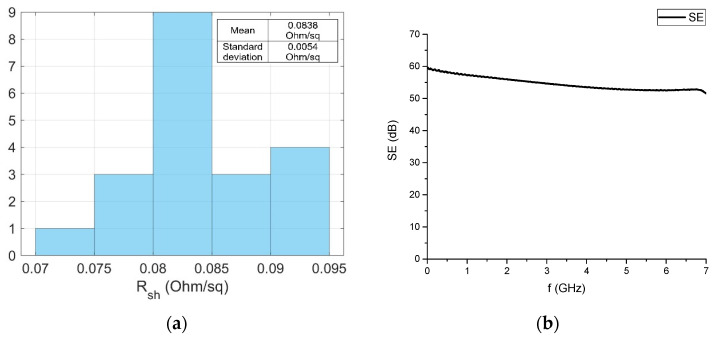
Sheet resistance distribution (**a**) and frequency dependence of the shielding efficiency (**b**) of the test sample.

The sheet resistance of the manufactured sample was (0.084 ± 0.016) Ω/sq. Measurements were taken at 22 different points on the sample surface (Figure 8a).

The frequency dependence of the SE is shown in Figure 8b. The average SE value in the frequency range of 10 MHz to 7 GHz is 54.6 dB. The horizontal behavior of the SE spectrum indicates the absence of significant metallization defects.

**Figure 9:**
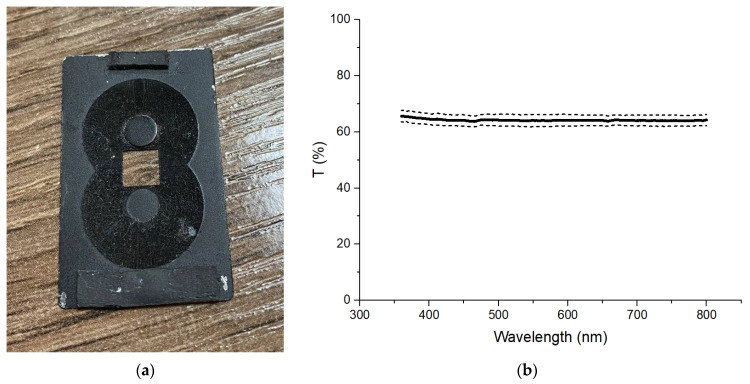
Photo of measuring equipment (**a**) and transmission spectrum of the test sample in the visible wavelength range (**b**).

The transmission spectrum measurements were taken at eight different points on the sample surface using a specially designed fixture (Figure 9a). The average, maximum and minimum transmission spectrums of the test sample in the visible wavelength range are shown in Figure 9b. The light transmittance coefficient calculated for the wavelength range from 380 to 780 nm following GOST R 54164-2010 is 64.1%.

## 5. Discussion

**Figure 10:**
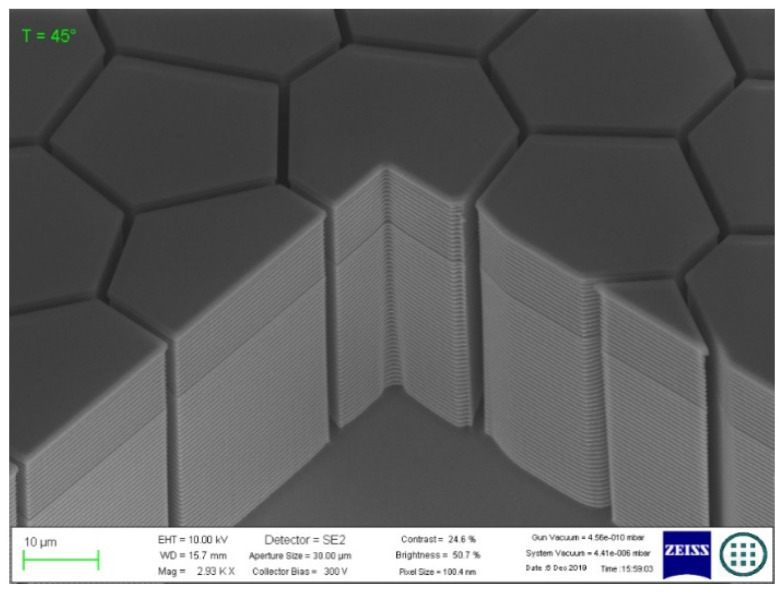
SEM images of the Si sample with grooves formed in its surface using the Bosch process with a high aspect ratio.

The deviation from the groove walls’ geometry due to the technological peculiarities of quartz etching led to a width increase from 1.5 to 4 µm at the groove entrance. This increased the surface area of the conductive grid paths and decreased the light transmission coefficient from 90% (according to the simulation results) to 64%. In order to increase this parameter, further development of the plasma chemical etching process of a transparent substrate is required. Notably, no such problems are encountered, for example, when forming grooves on Si substrates: it is possible to produce grooves with a high aspect ratio without distorting the geometry using the Bosh process, but such samples are only transparent in the IR range (Figure 10).

Transparency can also be increased by applying antireflective coatings based on metal oxides to the sample surface. These coatings will protect the metal mesh structure from oxidation in addition to improving optical properties.

The values of SE and sheet resistance obtained on the experimental sample were close to the calculated values. The silver synthesized in the grooves using the silver mirror reaction had a porous structure, and its electrical conductivity differed significantly from that of a monolithic material. The low value of the sheet resistance of the resulting microgrid (0.084 ± 0.016 Ω/sq) and, consequently, the high SE coefficient were caused by an increase in the cross-section of the conductors when the geometry of the conducting lines was distorted. Adjustment of the technology used for filling the grooves accompanied by an increase in the density of the formed channel can significantly increase the values of these parameters.

## 6. Conclusions

We developed a technology for producing transparent conductive electrodes. The technology consists of forming grooves in a transparent wafer by photolithography methods, followed by reactive ion plasma etching and its metallization by chemical deposition with a silver mirror reaction (Tollen′s reagent). This technology was chosen due to the high availability of necessary technological equipment and the verified technological regimes. Notably, methods of nanoimprint lithography and multilayer electrohydrodynamic (EHD) printing have considerable potential with the development of the methods for the creation of serial technology micro-mesh transparent electrodes with a large surface area.

The experimental sample was prepared according to developed technology with a sheet resistance of 0.09 Ω/sq and a visible light transmittance of more than 60%. The development of technology will allow for upgrading the functional characteristics of the prepared samples. In particular, the verticality of the groove wall needs to be improved for transparency improvement. The grade of the metal filling and the density of the metal need to be increased for enhancing the SE and decreasing sheet resistance. The first problem can be solved by reworking the reactive ion plasma etching of transparent wafers. The second task may be achieved using a sintering process for the prepared samples.

The proposed approach to the design of optically transparent electrodes may be effective for multiple scientific and industrial applications including acousto-optics, photoacoustics, lithography, radio shielding applications, etc.

## Data Availability

Not applicable.
